# Supplementary material for: Does Informal Education Training Increase Awareness of Anaphylaxis among Students of Medicine? Before-After Survey Study
Source: Int J Environ Res Public Health. 2021 Aug 1;18(15):8150. doi: 10.3390/ijerph18158150 (PMC8346085; doi:10.3390/ijerph18158150)
Supplement: Supplementary file 1 [file ijerph-18-08150-s001.zip › ijerph-1243072-supplementary.pdf]

Questionnaire about anaphylaxis:

Dear colleagues, as members of the Student Scientific Circle at the Clinic of Pediatrics, Gastroenterology, Allergology and Nutrition of Medical University of Gdańsk, we would like to invite you to participate in our research project. We will ask you to complete a short survey about anaphylaxis, then we will give you some practical directions on first aid in case of anaphylactic shock (please do not be afraid, we will use demonstration AAIs (adrenaline auto-injectors) during the training (without a needle). Finally, we will ask you to complete a short survey again. This will take 15 minutes only. By completing this questionnaire (completely anonymously) you agree to participate in the study. We would like to invite you to take part in the research and thank you for your time.

Gender: F/M

Year of study: 1/2/3/4/5/6

Introductory questions:

1. Are you diagnosed with allergy?

a) yes

b) no

2. Have you ever seen anaphylactic shock?

a) yes

b) no

3. Have you ever used AAI (adrenaline auto-injector)?

a) yes

b) no

General questions:

4. What is an anaphylactic shock?

a) a mild non-life-threatening allergic reaction

b) a severe, life-threatening hypersensitivity reaction which may occur with decreased blood pressure

c) a severe, life-threatening hypersensitivity reaction that occurs with increased blood pressure

d) insufficient activation of the immune system in response to allergens

e) allergic reaction to food allergens

5. What can cause anaphylactic shock?

a) peanuts, eggs, crustaceans, antibiotics

b) hazelnuts, soybeans, bee venom, latex

c) peanuts, fish, citrus, physical exertion

d) milk, painkillers, exercise, horse hair

e) all correct

6. What are the symptoms of anaphylactic shock?

a) shortness of breath, swelling of the tongue and eyelids, hives, increased blood pressure,

b) shortness of breath, throat tightness, palpitations, dry skin

c) shortness of breath, palpitations, paleness and wet skin, decreased blood pressure, loss of consciousness

d) shortness of breath, redness, ankle swelling, dry mucous membranes, fever

e) shortness of breath, petechiae, decrease in saturation, convulsions

Specific questions:

7. The first-line drug (s) in anaphylactic shock are:

a) antihistamines, e.g. clemastine

b) steroids, e.g. hydrocortisone

c) adrenaline

d) beta-2 mimetics, e.g. salbutamol

e) all correct

8. What dose of the drug should be given?

a) adults: 1 mg, children: 0.3 mg/kg

b) adults: 1 mg, children: 1 mg

c) adults: 0.3 mg-0.5 mg, children: 0.01 mg/kg

d) adults: 2.5 mg, children: 0.25 mg

e) adults: 10 mg, children: 1 mg

9. What is the route of administration?

a) inhalation

b) intravenously

c) subcutaneous

d) intramuscularly

e) oral

10) What should you do if you see anaphylactic shock?

a) take history, give medicine, carry out a physical examination

b) carry out a physical examination, administer the drug, go out and draw breath

c) provoke vomiting, call an ambulance

d) give the medicine, lay the patient someone down in at safe safe position, call an ambulance

e) try to calm the patient, go out into the fresh air, give something sweet

11) What is the shelf life of AAI?

a) 1 month

b) 6 months

c) 12 months

d) 2 years

e) 3 years

12) Would you be able to use AAI in case of shock?

a) yes

b) no

13) Do you think the training was useful?

a) yes

b) no
